# Determination of Antioxidant Capacity, Phenolics and Volatile Maillard Reaction Products in Rye-Buckwheat Biscuits Supplemented with 3β-d-Rutinoside

**DOI:** 10.3390/molecules24050982

**Published:** 2019-03-11

**Authors:** Małgorzata Starowicz, Georgios Koutsidis, Henryk Zieliński

**Affiliations:** 1 Department of Chemistry and Biodynamics of Food, Division of Food Science, Institute of Animal Reproduction and Food Research of the Polish Academy of Sciences, Tuwima 10, P.O. Box 55, 10-748 Olsztyn 5, Poland; h.zielinski@pan.olsztyn.pl; 2 Department of Applied Sciences, Faculty of Health and Life Sciences, Northumbria University, Newcastle upon Tyne NE1 8ST, UK; georgios.koutsidis@northumbria.ac.uk

**Keywords:** volatiles, rutin, antioxidant capacity, pyrazines, furans, sensory evaluation, HS-SPME/GC–MS

## Abstract

The Maillard reaction (MR) is responsible for the development of color, taste and aroma in bakery products though the formation of numerous aroma compounds such as pyrazines, pyrroles and aldehydes, nonvolatile taste active compounds and melanoidins. In this article, we investigate the effect of quercetin 3β-D-rutinoside (rutin) supplementation, at the level of 5–50 mg per 100 g, of rye-buckwheat biscuits on the formation of phenolics and volatile Maillard reaction products (MRPs) such as pyrazines, furfuryl alcohol and furfural, determined by headspace solid phase microextraction followed by gas chromatography–mass spectrometry (HS-SPME/GC–MS), in addition to the effect on the antioxidant capacity. The study confirmed that rutin was stable under baking conditions as showed by its content in rye-buckwheat biscuits. Supplementation of biscuits with increasing amounts of rutin resulted in the progressive increase of total phenolics and antioxidant capacity measured by DPPH and OxHLIA assays, but it had no effect on their sensory quality. From the eighteen compounds identified by HS-SPME/GC–MS in the volatile fraction of biscuits were quantitated as a compounds-of-interest: methylpyrazine, ethylpyrazine, 2,3-; 2,5- and 2,6-dimethylpyrazines, as well as furfural, furfuryl alcohol and hexanal. The rutin supplementation of biscuits might be one of the factors to influence the formation of both desirable volatile compounds and undesirable toxic compounds. In conclusion, this study indicates for the significant role of polyphenols on the formation of volatile compounds in biscuits with possible future application in the development of healthy bakery products with high antioxidant capacity.

## 1. Introduction

Generally, compounds that contribute to the overall aroma of the foodstuff are thermally generated through Maillard reaction (MR), lipid oxidation or caramelization [1]. Flavor active Maillard reaction products (MRPs) can be sorted into four groups: pyrazines/pyrroles/pyrrolidines, aldehydes, thiazoles/thiazolines/thiophenes and oxazoles and furans. The main pathway to pyrazines’ formation in foods is through the condensation of amino-carbonyls from the hydrolysis of the Amadori compounds. Pyrazine derivatives might be also formed in a reaction between ascorbic acid and amino acids as described by Mariotti et al. [2] and Ai Nong et al. [3]. Moreover, pyrazines were determined as predominant components of volatile profile in products after enzymatic treatment [4]. Pyrazines are important contributors of flavor of heated foods, namely beef, toasted barley, cocoa, coffee, peanuts, popcorn, potato chips and rye crisp bread and have been investigated for their characteristic nutty and roasted odor notes [5]. In advanced stages of MR, undesirable compounds such as furfurals can be found in bread, biscuits, marmalade, honey and breakfast cereal samples. Furans are formed during aldol condensation of acetaldehyde and glycolaldehyde after cyclization and dehydration steps, degradation of monosaccharides, non-oxidative pyrolysis of ascorbic acid though the 2-deoxyaldotetrose moiety or through lipid peroxidation via 4-hydroxy-2-butenal, a known decomposition product from polyunsaturated fatty acids [6]. According to studies with animals exposed to high furan doses, the IARC categorized furans as belonging to a group of possible carcinogens to humans. 

An analysis of aroma composition of buckwheat grains has been recently conducted, where salicylaldehyde was identified as a characteristic component of buckwheat aroma [7]. Among the compounds of interest which contributed to buckwheat aroma, methylpyrazine, 2,5-dimethylpyrazine, furfural and furfuryl alcohol were also identified. Some studies were also focused on aroma profiles of buckwheat honey [8]. The researchers declared that furfural, with an odor of sweet almonds, and methylbutyraldehyde, with pungent, sweet, malty and burnt cocoa smell, can be specific markers of buckwheat honey [8]. Wolski et al. [9] were first to employ GC–MS with solid phase microextraction as a method to determine the aroma of buckwheat honey. According to our state of knowledge, not many investigations on volatile MRPs formation in buckwheat-based bakery products were held.

The headspace solid phase microextraction coupled with gas chromatography–mass spectrometry (HS-SPME/GC–MS) method was established as a new approach for use in determining volatile compounds of different origin, such as aldehydes and alcohols, in plant and cereal products such as in breakfast cereal [10] and corn flakes [11]. The main advantages of this technique are the simplicity of implementation, elimination of extraction solvents and reduction in sample processing time. SPME has been successfully applied in the analysis of volatile and semivolatile compounds from differential food products [12]. 

Some studies reported a significant influence of polyphenols derived from natural sources, on Maillard reaction products (e.g., acrylamide and furan derivatives formation) [13]. Moreover, Totlani and Peterson [14] reported that epicatechin addition significantly reduced the quantities of furfural and furfuryl alcohol in MR model systems. In further studies, Klensporf and Jeleń [10] noticed that the addition of a high-antioxidant extract from raspberry seeds to breakfast cereal inhibited the formation of undesirable Maillard-type aroma compounds. A positive effect of raspberry seeds in limiting the autoxidation process of muesli was observed. Budryn et al. [15] also observed a significant increase of pyrazines after chlorogenic acid (extracted from green coffee) application. 

The aim of this study was to investigate the role of rutin as well as phenolic compounds on the formation of volatile compounds in rye-buckwheat biscuits—with possible future application in the development of healthier biscuits with high antioxidant capacities and a greater control of the formation of both desirable volatile compounds and undesirable toxic compounds. To achieve this goal, evaluation of the rutin content, antioxidant capacity (measured by DPPH and OxHLIA assays), determination of volatile MRPs by HS-SPME/GC–MS and sensory analysis of rye-buckwheat biscuits enriched with rutin were addressed in this study.

## 2. Results and Discussion

### 2.1. Total Phenolic Compounds (TPC) and Rutin Contents, and Antioxidant Capacity of Rye-Buckwheat Biscuits

Flours, buckwheat honey and spices are the main sources of phenolic compounds in rye- buckwheat biscuits. The TPC of control biscuits was 0.90 mg GAE/g DW, while in buckwheat biscuits and biscuits at low, medium, and high rutin addition (RBB-L, RBB-M, and RBB-H, respectively), the TPC ranged from 2.22 to 22.39 mg GAE/g DW (Table 1). 

The addition of buckwheat flour was found to have an influence in increasing TPC content 2.5-times (from 0.90 in control to 2.22 mg GAE/g DW in rye-buckwheat biscuits (RBB)). The highest TPC content was measured in RBB-M and RBB-H samples. TPC increased in RBB-M and RBB-H about 12 and 25-times, respectively, in comparison to control biscuits; and 5- and 10-times in comparison to RBB. Selimović et al. [16] reported significantly lower TPC content (0.52 mg GAE/g DW) in wheat-wholegrain buckwheat bread (flour mixed ratio 70:30%, w/w) than in rye-buckwheat biscuits. Moreover, TPC content in RBB sample was slightly higher than in wheat-buckwheat bread (2.65 mg GAE/g DW) [16]. This may also be due to the fact that in the rye-buckwheat biscuit recipe buckwheat honey and spices highly contribute to the overall amount of TPC. 

Among phenolic compounds, buckwheat contains high amounts of rutin in comparison to other cereal and pseudo cereal grains [17]. The concentration of rutin was determined in an 80% (*v*/*v*) methanol extract of rye-buckwheat biscuits with the HPLC method. The results are presented in Table 1. This measurement is necessary to check the adequate rutin amount in final product after baking process. It is known that rutin is not heat-stable and that it can degrade in the baking process, probably to quercetin [18]. Sakač et al. [19] declared that around 40% of rutin degraded during baking in gluten free breads. Sofic et al. [18] established that rutin content in buckwheat flour (*Fagopyrum esculentum* M.) is about 120 µg/g DW whereas Sakač et al. [19] reported that content of rutin in light buckwheat flour is at the level of 87 µg/g DW. Supplementation of the biscuit formula by different amounts of rutin resulted in a progressive increase in the rutin content in cakes RBB-L, RBB-M and RBB-H. Most significantly, rutin content increased in RBB-M and RBB-H samples, 4-times and 9.6-times, respectively. 

The functional properties of rye-buckwheat biscuits were evaluated with in-vitro antioxidant assays. The values of antioxidant properties results, expressed as DPPH and FRAP values, are summarized in Table 2. The DPPH values of rye-buckwheat varied from 2.84 (RB) to 3.22 µmol Trolox/g DW (RBB-H), whereas the control biscuit extract scavenged DPPH radicals at a level of 2.65 µmol Trolox/g DW. The buckwheat flour addition at the 30% level in the formulae resulted in significant differences (*p* < 0.05) in the antioxidant capacity. Similar results have been presented in previous studies by Jan et al. [20], who reported increasing DPPH values according to increasing incorporation of buckwheat flour. Neither low nor medium rutin addition had a significant influence on increasing antioxidant status of rye-buckwheat biscuits. The highest DPPH scavenging ability was observed in RBB-H, in which the high level of rutin addition increased at 22% and 13% in comparison to control and RBB samples, respectively. The scavenging ability of rye-buckwheat biscuits against DPPH radical was not as high as for wheat-buckwheat (70:30, *w*/*w*) bread [16]. This might be due to the high temperature used during the rye-buckwheat biscuits baking process, which might enable the degradation of flavonoids. Consequently, OxHLIA results ranged from 10.67 (control) and 11.39 (30% buckwheat addition) to 12.34 µmol Trolox/g DW. The OxHLIA method is based in principle on the inhibition of free radical-induced membrane damage in erythrocytes by antioxidants [21]. The incorporation of 30% of buckwheat flour significantly increased the antioxidant potential of rye-buckwheat biscuits, almost 7% in RBB. Then, no significant change was observed after low and medium rutin addition (RBB-L and RBB-M). Only the addition of a high amount of rutin to biscuits increased their antioxidant potential, by 15 and 8% when compared RBB-H to C and RBB, respectively. The antioxidant potential of RBB-H samples was on the same level as orange and strawberry juices measured using AAPH-induced cell hemolysis method by Prior et al. [22]. 

High correlation coefficients between rutin vs. TPC contents, rutin vs. DPPH and rutin vs. OxHLIA were calculated (r = 0.997; 0.950; 0.917). Moreover, the strong correlation was noticed between TPC content and antioxidant activity (r = 0.953; 0.916 for DPPH and OxHLIA, respectively). This is in accordance with Yang et al. [23], who reported that rutin is “a powerful free radical inhibitor or scavenger” using DPPH assay. Our results also pointed out that there is a strong correlation between DPPH and OxHLIA assays (r = 0.988).

### 2.2. Sensory Evaluation

**Figure 1:**
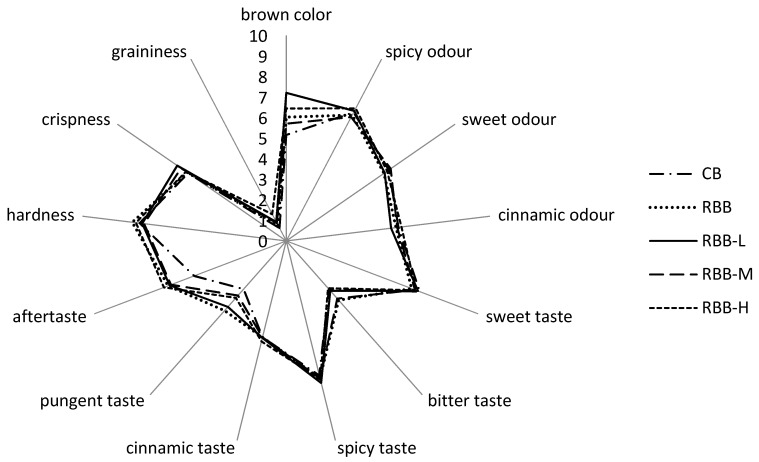
Aroma profiles of control (CB), rye-buckwheat biscuit (RBB) and RBBs with low, medium and high rutin addition (RBB-L, RBB-M, RBB-H, respectively).

The influence of buckwheat flour and varying doses of rutin addition is presented in Figure 1. After addition of buckwheat flour, the overall quality of RBB (5.8) decreased in comparison to control ones (6.4). These findings are in agreement with Bajleet et al. [24], who reported that the incorporation of buckwheat flour decreased consumer acceptance of buckwheat based-product. The overall quality score of rye-buckwheat biscuits was established at 5.7, 5.9 and 6.2 for RBB-L, RBB-M and RBB-H, respectively. A score at a level of 6.2 was also achieved for the buckwheat biscuits of Bajleet et al. [24] with 30% buckwheat incorporation. Moreover, the positive acceptance of buckwheat product was proven by Chlopicka et al. [17]. It can be said that rutin addition didn’t significantly influence the acceptance score of rye-buckwheat biscuits. In detail, the 13 sensory descriptors were evaluated by panelists assessing: color, aroma, taste and texture of rye-buckwheat biscuits. The statistically significant difference was observed in color acceptance (*p* < 0.0001). Therefore, the acceptability of color was lowest for control while the best score was achieved for RBB-L. The 30% incorporation of buckwheat flour significantly increased the color acceptance from 5.13 to 6.03 (*p* < 0.0001). A similar observation about color of buckwheat-based bread was presented by Lin et al. [25], who reported that buckwheat improved breads’ color due to high phenolic content inhibiting the browning compounds’ formation via Maillard reaction during baking. Moreover, the color of rye-buckwheat biscuits is more attractive than for rice-buckwheat cookies with the same amount of buckwheat flour [19].

In case of odor and taste, only for aftertaste significant difference was noticed between control and rye-buckwheat biscuits (*p* < 0.001). The 30% buckwheat flour incorporation influenced, while rutin addition didn’t affect the aftertaste impression. The pungent and bitter taste of rye-buckwheat biscuits can be related to high concentration of rutin and catechin in buckwheat flour [26]. Bitter taste increased significantly (*p* < 0.0001) in rye-buckwheat biscuits with medium and high rutin addition, RBB-M and RBB-H, respectively. To avoid problem with strong bitter taste of buckwheat, some researchers are working on non-bitter type of buckwheat, which might in future increase consumption of buckwheat [27]. The high contribution to panelists acceptation might have been associated with sweet and spicy taste. For sweet taste, the addition of buckwheat honey and sugar are responsible, whereas spicy taste is related to spice mix incorporation to biscuits recipe. Attractiveness of buckwheat biscuits is probably also elaborated by cinnamon taste and odor, which are linked to cinnamon presented in spice mix. 

Regarding textural properties, incorporation of buckwheat flour negatively influenced the hardness of biscuits in comparison to control, however the difference was not statistically significant. The rye-buckwheat biscuits were harder to bite than rice-buckwheat cookies as was studied previously by Sakač et al. [19]. The worse score for hardness in our experiment might be due to different rheological properties for rye dough. However, in latest studies by Sakač et al. [19], panelists declared also a high score for crispness of rye-buckwheat biscuits. The graininess of rye-buckwheat biscuits, which describes unpleasant mouth feeling, could be attributed to the coarse granularity of buckwheat flour. Ahmad et al. [28] noted very good textural properties of cookies with green tea addition, however in our case usage of gluten-free buckwheat flour is a main problem to achieve high rheological scores. 

### 2.3. Volatile Maillard Reaction Products Formation

In this study, GC–MS with SPME technique was used to identify the volatile compounds of rye-buckwheat biscuits. The results for identified volatiles are shown in Table 2 and Table 3. The main identified volatile compounds belong to aldehydes, terpenes and pyrazines. 

**Aldehydes and ketones**. The 2-methylpropanal and 3-methylbutanal are the volatile MRPs, formed via compounds of Strecker degradation. The content of 3-methylbutanal significantly increased in RBB and RBB-H samples (*p* < 0.05). The amount of 2-methylpropanal increased after buckwheat flour substitution but then decreased in RBB-H (*p* < 0.05). The amount of 2-methylpropanal is almost 12-times higher than in sponge cake [1]. It may be due to the fact that in our recipe we used a high amount of butter instead of oil. It is known that 3-methylbutanal is responsible for malty aroma of buckwheat honey. Another aldehyde that has been found in the volatile profile of rye-buckwheat biscuits was (E)-cinnamonaldehyde. It can be considered that cinnamon from spice mix is a main source of (E)-cinnamonaldehyde in biscuits and is responsible for their pleasant sweet aroma [29]. The concentrations of pentanal were considerably greater in samples with rutin supplementation. The peaks of pentanal were observed to be double the height in RBB-H, in comparison to control (*p* < 0.05). Paradiso et al. [11] observed that pentanal concentration decreased in model cookies after tocopherol addition, however after application of rosemary extract the amount of pentanal significantly increased. Hexanal amount decreased by 12% in biscuits with 30% buckwheat flour addition, then another 1.75-times and 2.40-times in RBB-L and RBB-H, respectively in comparison to RBB (Table 3) (*p* < 0.05). Our findings are in accordance to Klensporf and Jeleń [10], who evaluated that raspberry seeds addition decreased formation of hexanal in cereal flakes. The hexanal concentration in rye-buckwheat biscuits is 10-times higher than that identified in flakes. This might be due to the fact that higher temperature was used in biscuits baking procedure. Hexanal was recognized as a one of compound with the highest concentration and contribution to the buckwheat aroma [7]. Moreover, hexanal is known as important indicator of food samples quality after longer storage period. Negative correlation coefficients between hexanal vs. rutin and TPC contents; and hexanal vs. DPPH and OxHLIA were calculated (r = −0.77; −0.81; −0.88 and −0.86). Amongst ketones 2-heptanone and 2-nonanone were determined. Ketones are mainly formed in lipid oxidation process. The highest amount of 2-heptanone was determined in RBB-H. Our observations are similar to Paradiso et al. [11], who reported that a mix of tocopherol and ascorbic acid significantly increased the peak area of 2-heptanone. It can be said that rutin supplementation might positively influence then quality of rye-buckwheat biscuits during storage.

**Terpenes** are a varied group of volatile metabolites synthetized generally by plants [30]. A great number of terpenes were previously described in spices, which are ingredients in the spice mix used in the biscuit recipe: α-pinene in nutmeg, anise, coriander, pepper and fennel, β-myrcene in nutmeg and coriander, 1,8-cineole (eucalyptol) in cinnamon, D-limonene in anise, pepper and fennel, and caryophyllene in cinnamon, clove and pepper [31]. However, α-pinene and D-limonene were also found in buckwheat aroma profile [7]. Not significant changes were observed in terpenes identification, only caryophyllene increased 1.50-fold in RBB samples and in samples with rutin addition (*p* > 0.05).

**Pyrazines**. Pyrazines are volatile compounds formed via Maillard reaction and have characteristic nutty and roasty aroma. Increasing rutin addition from 5 mg up to 50 mg per 100 g of biscuits caused an increase in pyrazine content (*p* < 0.05). The methylpyrazine content increased 1.7-times in comparison to control and RBB. Then, in 2,5- and 2,3-dimethylpyrazine no significant difference after buckwheat flour addition was observed (*p* > 0.05). Therefore, 2,3-dimethylpyrazine increased by around 16% and 2,5-dimethylpyrazine 2-times in RB-H. 2,6-methyl- and ethylpyrazines, were not present in control and RBB samples. They appeared in RBB-L and their concentrations significantly increased in biscuits with medium and low rutin additions (*p* < 0.05). These findings are in accordance to Adams and De Kimpe [32] and Ai-Nong et al. [3], who reported that pyrazine formation can be induced by the addition of ascorbic acid (strong antioxidant). Ascorbic acid can react with amino acids and form final stages of different pyrazine derivatives. Also, Totlani and Peterson [14] demonstrated that the addition of another antioxidant (epicatechin) influenced the higher amount of methylpyrazine present. 

**Furans.** Among furan derivatives, furfural and furfuryl alcohol were identified in rye-buckwheat biscuits (Table 3). It is known that high concentrations of furfural in bakery products may cause allergic skin reactions in consumers and furfuryl alcohol can cause a bitter taste of baking products. Furfural was identified as a main volatile component in buckwheat honey [8]. Ozolina et al. [33] established that baking bread for 30 min is the appropriate time to avoid significant amount of furfural. The positive effect of rutin on reducing furan derivatives in rye-buckwheat biscuits was observed. Firstly, buckwheat flour supplementation and then rutin in RBB-H decreased furfural concentration (*p* < 0.05). The concentrations of furfural and furfurol in rye-buckwheat biscuits were about 10-times and 12-times lower, respectively than in cookies described by Kowalski and Lukasiewicz [34]. Some previous researchers demonstrated that antioxidants like caffeic acid [13] or epicatechin [14] limited the formation of furfural. The results of rutin and its antioxidant properties were shown to (Table 4) display a negative correlation coefficient calculated between formation of furfural and furfuryl alcohol and rutin, TPC contents, DPPH, OxHLIA assays (r = −0.66, −0.70, −0.84, −0.85; r = −0.61, −0.65, −0.79, −0.80).

**Other volatiles.** The other compounds which weren’t classified previously but determined in rye-buckwheat biscuits are 4-hydroxybutanoic acid and estragole. In spices such as anise and fennel, estragoles highly contribute to their overall aroma [28].

## 3. Materials and Methods

### 3.1. Standards and Reagents

Rutin (quercetin-3-rutinoside), 2,2-diphenyl-1-picrylhydrazyl (DPPH), 6-hydroxy-2,5,7,8-tetramethylchroman-2-carboxylic acid (Trolox), sodium carbonate dehydrate (Na_2_CO_3_), sodium chloride (NaCl), sodium dihydrogen phosphate, disodium hydrogen orthophosphate, iron (III) chloride hexahydrate (FeCl_3_•6H_2_O), Folin–iocalteu reagent, furfural, furfuryl alcohol, methylpyrazine, ethylpyrazine, 2,3-dimethyl-, 2,5-dimethyl- and 2,6-dimetylpyrazine, and methanol were purchased from Sigma (Sigma Chemical Company, St. Louis, MO, USA). 2,4,6-tri(2-pirydyl)-s-tiazine (TPTZ) and 2,2′-azobis(2-methylproprionamidine) dihydrochloride (AAPH) was provided by Fluka (Germany). Sheep blood in Alsevers solution was purchased from Oxoid Limited.

### 3.2. SPME Fiber Conditioning

For HS-SPME, 50/30 μm divinylbenzene/Carboxen/polydimethylosiloxane (DVB/Carboxen/PDMS) stable flex^TM^ fiber coating was purchased from Supelco (Bellefonte, PA, USA). Before analysis, fiber was conditioned at 250 °C for 30 min in order to remove contaminants and to stabilize the solid-phase. The fiber was chosen in experimental procedure (data not shown).

### 3.3. Rye-Buckwheat Biscuits Preparation Formula

The biscuit making process involved dough preparation by mixing flours, honey and sugar. Light buckwheat flour from unhusked common buckwheat groats (*Fagopyrum esculentum* M.) was bought in shop with healthy food. Rye flour (type 720), buckwheat honey (*Fagopyrum esculentum* M., from Warmia and Mazury region, Poland) and other typical bakery ingredients were supplied from local market in Olsztyn, Poland.

The formulation of rye-buckwheat biscuits enriched with rutin are shown in Table 5. The recipe of rye-buckwheat ginger cakes was modified by adding low, medium and high amounts of rutin to the mixture of flours: 5 mg, 25 mg and 50 mg per 100 g of biscuits, respectively. The prepared dough was cut into 0.5-cm thick discs of 5.5 cm diameter and baked at 180 °C for 18 min in an electric oven (DC-32E, Sveba-Dahlen, Fristad, Sweden). The biscuits were freeze-dried, ground into powder, sieved through a 60-mesh screen and finally stored at −20 °C until analyzed. Some biscuits were stored as a whole in plastic bags for sensorial analysis. 

### 3.4. Determination of Rutin and TP Contents, and Antioxidant Capacity of Rye-Buckwheat Biscuits

An aliquot of 0.10 g of powdered biscuit samples was extracted with 1 mL of 80% (*v/v*) methanol. The mixture was sonicated, vortexed each for 30 s, repeated three times and centrifuged for 5 min (5000× *g* at 4 °C). That step was repeated five times and the supernatants were collected into 5-mL flasks. The final extract concentration was 20 mg/mL. Extraction of each sample was performed in triplicate. The concentrated 80% methanol extracts from rye-buckwheat biscuits for rutin and total phenolic content (TPC), and antioxidant capacity determinations were used.

The content of rutin in rye-buckwheat biscuits was determined with HPLC (Shimadzu, Japan) with UV detector (SPD-10A) set up 330 nm as it was recently described by Zielińska et al. [35]. For quantitative analysis, rutin standard was prepared in triplicate at five concentrations within the range of 1.0–40 μM. All solutions were filtered through a 0.45 μm nylon membrane before use. The results were expressed in μg/g of dry weight (DW). 

The TPC content and antioxidant capacity by DPPH of rye-buckwheat biscuits with increasing rutin addition was carried out as previously described in detail by Magalhães et al. [36]. The OxHLIA method was performed as described by Takebayashi et al. [19] to measure antioxidant activity in-vitro. Both measurements were performed in microplate reader (Infinite M1000 Pro Multimode Microplate Reader, Tecan GmbH, Grödig, Austria), using transparent 96-well microplates for absorbance readings (Porvair, TK Biotech). TPC content was expressed in mg of Gallic acid equivalents (GAE)/g of DW whereas results of DPPH and OxHLIA methods were expressed as μmol Trolox/g of DW.

### 3.5. Sensory Evaluation of Rye-Buckwheat Biscuits

The attributes related to the appearance, odor, taste and texture of rye-buckwheat biscuits with spices were selected and thoroughly used to during profiling procedure. Sensory characteristics and overall quality of ginger cakes were evaluated according to international unified standards. A 6-member trained panel judged ginger cakes in a 10-point scale (0—for weak, 10—for very good) using Quantitative Descriptive Analysis (QDA) to determine differences between each type of biscuits. The description of sample preparation and standardized procedure of sensory evaluation were in details presented by Zieliński et al. [37].

Overall acceptability of each sample was evaluated in relation to the sensory preferences on the basis of overall appearance, aroma, taste and texture, in a ten-point hedonic scale, where: 0 = not accept, and 10 = fully accept. The profiling analysis of all samples was run in duplicate (two series) proceeded by introduction session. 

### 3.6. Volatile Maillard Reaction Compounds Analysis by HS-SPME/GC-MS

About 2 g of powdered rye-buckwheat biscuits were transferred into 22-mL headspace vials. The vials were sealed air-tight with a silicone/polytetrafluoroethylene (PTFE) septum. An aliquot of 2.5 mL of sodium chloride solution (25%) was added to each vial before capping and heating. A GC-MS method developed previously by Koutsidis et al. [38], was employed to quantitatively determine volatile compounds in the biscuits. The vials were then placed onto a COMBIPAL autosampler (CTC Analytics, Zwingen, Switzerland) coupled to an Agilent 7890A GC (Agilent Technologies, Colorado Springs, USA) and a BenchTOF-dx mass spectrometer (ALMSCO International, Llantrisant, UK).

HS-SPME of the preheated samples (40 °C, 5 min) was performed under agitation for 1 min at 500 rpm using a 50/30 μm DVB/Carboxen/PDMS stable flex^TM^ fiber (Supelco, Bellefonte, PA, USA), followed by desorption (5 min) at 250 °C onto a 60 m DB-WAX capillary column (0.25 mm i.d. −0.25 μm film thickness). The initial oven temperature was set at 40 °C, held for 5 min, increased to 200 °C at 4 °C min^−1^, held for 1 min, and finally increased to 260 °C at 8 °C min^−1^, held for 5 min. The helium flow rate was maintained constant at a flow rate of 1 mL min^−1^. For quantification purposes, an external calibration method was used. Pyrazine standard solution was prepared by weighing 50 mg of pure standards of methylpyrazine, ethylpyrazine, 2,3-, 2,5- and 2,6-dimethylpyrazine in a 25 mL volumetric flask and make up the rest of the volume with de-ionized water. The 500 mL of standard solutions were added into the vial with 2 g of rye flour and 2.5 mL sodium chloride solution. All analyses were carried out in triplicate injection. Selected ions were used for quantification of the individual components. Compound identification was carried out by injection of commercial standards, by spectra comparison using the Wiley Registry 7th Edition Mass Spectral Library (Wiley and Sons Inc., Weinheim, Germany) and the National Institute Standards and Technology (NIST) 2005 Mass Spectral Library and by calculation of linear retention indexes (LRI) relative to a series of alkanes (C6–C20).

### 3.7. Statistical Analysis

The results of the chemical analyses are given as the means and the standard deviation of three independent measurements. Statistical one-way analysis of variance (ANOVA) using Fischer test was performed. The significance level was set at *p* < 0.05. The correlation test between rutin and TPC content, antioxidant ability and volatile MRPs formation was performed and the Pearson correlation coefficients were calculated. Statistical analyses were performed using software package (StatSoft Inc., v. 7.1, Tulsa, OK, USA).

## 4. Conclusions

The total phenolic content, rutin, volatile MRPs and antioxidant capacity were determined in rye-buckwheat biscuits supplemented with rutin. This study confirmed that rutin was stable under baking conditions and resulted in the progressive increase of total phenolics and antioxidant capacity measured by DPPH and OxHLIA assays. However, no effect on the biscuits’ sensory quality was observed. The good acceptability score was achieved for biscuits with rutin supplementation, despite the bitter taste appearing after buckwheat flour incorporation. The HS-SPME/GC-MS method allows to control the formation of both desirable volatile compounds and undesirable toxic compounds. Addition of rutin to rye-buckwheat biscuit recipes induced the concentration of pyrazine derivatives. In the other hand, the formation of furfural and furfurol were significantly inhibited in rye-buckwheat biscuits after rutin application. These results were firmly correlated with rutin, total phenolics and antioxidant capacity measurements. According to obtained results, rye-buckwheat biscuits with medium (RBB-M) and high rutin addition (RBB-H) can be defined as functional food. 

It can be concluded that this study indicates a significant role of rutin in the formation of volatile compounds in rye-buckwheat biscuits with possible future application in the development of healthy biscuits with high antioxidant capacity. Further study might focus on the influence of rutin on acrylamide formation in the aspect of food safety of bakery products.

## Figures and Tables

**Table 1:** Rutin and Total Phenolic Compounds (TPC) contents and antioxidant capacities of rye-buckwheat biscuits enriched with rutin as determined by DPPH and OxHLIA methods.

Biscuits Sample	Rutin [µg/g of DW]	TPC [mg GAE/g of DW]	Antioxidant Capacity [µmol Trolox/g DW]	
			DPPH	OxHLIA
CB	1.78 ± 1.78 ^e^	0.90 ± 0.03 ^e^	2.65 ± 0.01 ^c^	10.67 ± 0.06 ^d^
RBB	28.51 ± 3.84 ^d^	2.22 ± 0.09 ^d^	2.84 ± 0.04 ^b^	11.39 ± 0.02 ^c^
RBB-L	37.73 ± 6.59 ^c^	4.77 ± 0.35 ^c^	2.88 ± 0.04 ^b^	11.34 ± 0.03 ^c^
RBB-M	115.13 ± 55.74 ^b^	10.35 ± 0.38 ^b^	2.98 ± 0.05 ^b^	11.82 ± 0.01 ^bc^
RBB-H	273.72 ± 1.08 ^a^	22.39 ± 0.08 ^a^	3.22 ± 0.09 ^a^	12.34 ± 0.05 ^a^

Abbreviations: CB—control biscuits made of rye flour, RBB—rye-buckwheat biscuits made of rye and light buckwheat flour (70:30, w/w), RBB-L—rye-buckwheat biscuits made of rye and light buckwheat flour with low rutin addition (5 mg rutin/100 g of product), RBB-M—rye-buckwheat biscuits made of rye and light buckwheat flour with medium rutin addition (25 mg rutin/100 g of product), RBB-H—rye-buckwheat biscuits made of rye and light buckwheat flour with high rutin addition (50 mg rutin/100 g of product). DW: dry weight, GAE: Gallic acid equivalents. Data are expressed as means ± SD, each measurement was repeated in triplicate. Values in each column with different small superscript letters are significantly different (*p* < 0.05).

**Table 2:** Volatile compounds identified in control biscuits (CB), rye-buckwheat biscuits (RBB) and in biscuits with increasing rutin addition (RBB-L, RBB-M, RBB-H, respectively). The compounds were identified by comparing its mass spectrum and RI with data in the literature: tentative identification. Results are expressed as Peak Area ×10^6^.

Compound	RI ^a^_LIT_	RI ^b^	Odor Description	CB	RBB	RBB-L	RBB-M	RBB-H
Aldehydes and ketones								
2-methylpropanal	611	625	malty	13.91 ± 0.91 ^c^	24.90 ± 0.14 ^a^	19.81 ± 0.12 ^b^	9.48 ± 0.53 ^e^	10.76 ± 0.47 ^d^
2-methylbutanal	912	927	malty	347.36 ± 19.31 ^d^	591.31 ± 12.60 ^c^	631.33 ± 32.52 ^c^	967.80 ± 55.38 ^b^	1087.94 ± 37.53 ^a^
Pentanal	935	946	pungent	15.11 ± 0.39 ^b^	13.85 ± 0.52 ^c^	14.75 ± 0.51b ^c^	36.47 ± 0.04 ^a^	35.58 ± 0.05 ^a^
(E)-cinnamaldehyde	1631	1641	cinnamon	354.82 ± 13.00 ^a^	354.19 ± 23.66 ^a^	309.96 ± 46.71 ^a^	355.24 ± 40.40 ^a^	302.82 ± 51.19 ^a^
2-heptanone	1160	1175	soapy	11.62 ± 0.22 ^b^	8.01 ± 0.19 ^d^	9.60 ± 0.90 ^c^	12.73 ± 0.84 ^a^	14.05 ± 1.71 ^a^
2-nonanone	1280	1289	soap, green	7.69 ± 0.71 ^ab^	6.39 ± 0.44 ^c^	7.58 ± 0.16 ^b^	8.23 ± 0.37 ^a^	8.56 ± 0.16 ^a^
Terpenes								
α-pinene	1034	1039	rosin	0.45 ± 0.02 ^a^	0.40 ± 0.07 ^a^	0.41 ± 0.06 ^a^	0.38 ± 0.01 ^a^	0.41 ± 0.02 ^a^
β-myrcene	1176	1156	geranium, sweet	0.18 ± 0.02 ^a^	0.12 ± 0.04 ^a^	0.14 ± 0.02 ^a^	0.17 ± 0.07 ^a^	0.22 ± 0.02 ^a^
1,8-cineole	1224	1234	eucalyptus-like	26.50 ± 1.48 ^a^	27.38 ± 2.05 ^a^	20.67 ± 4.85 ^a^	21.59 ± 5.15 ^a^	27.98 ± 0.21 ^a^
D-limonene	1234	1244	citrus-like	22.02 ± 1.36 ^a^	23.12 ± 2.22 ^a^	29.61 ± 5.15 ^a^	26.21 ± 1.61 ^a^	23.15 ± 0.41 ^a^
Caryophyllene	1594	1604	wood, spicy	83.04 ± 6.05 ^c^	123.31 ± 14.10 ^ab^	124.66 ± 18.64 ^ab^	104.40 ± 9.89 ^b^	136.29 ± 10.79 ^a^
Other								
Estragole	1655	1666	licorice, anise	51.06 ± 5.11 ^a^	7.62 ± 1.86 ^b^	7.31 ± 0.28 ^b^	7.02 ± 0.13 ^b^	7.87 ± 0.37 ^b^

^a^ Retention indexes from literature: pherobase.org for DB-WAX. ^b^ Calculated retention index values relatively to the n-alkane ladder. Values are means ± standard deviation (n = 3). Values in each column with different small superscript letters are significantly different (*p* < 0.05).

**Table 3:** Volatile compounds identified in control biscuits (CB), rye-buckwheat biscuits (RBB) and in biscuits with increasing rutin addition (RBB-L, RBB-M, RBB-H, respectively). The compounds were identified by comparing its mass spectrum and RI with standard compounds. Results are expressed as ng g^−1^ DW.

Compound	RI ^a^_LIT_	RI ^b^	Odor Description	CB	RBB	RBB-L	RBB-M	RBB-H
*Aldehydes*								
hexanal	1084	1078	grassy, green	31,297 ± 1228 ^a^	27,495 ± 644 ^b^	15,753 ± 695 ^c^	15,812 ± 917 ^c^	11,405 ± 133 ^d^
*Pyrazines*								
methylpyrazine	1253	1266	nutty, cocoa	1002 ± 55 ^c^	1041 ± 11 ^c^	692 ± 65 ^d^	1318 ± 9 ^b^	1785 ± 73 ^a^
2,6-dimethylpyrazine	1308	1310	nutty, earthy	nd	nd	396 ± 26 ^c^	521 ± 30 ^b^	633 ± 29 ^a^
2,5-dimethypyrazine	1320	1329	nutty, roasty	518 ± 18 ^b^	530 ± 51 ^b^	484 ± 5 ^c^	1035 ± 46 ^a^	1055 ± 13 ^a^
ethylpyrazine	1330	1341	nutty, buttery	nd	nd	57 ± 4 ^c^	96 ± 6 ^b^	135 ± 8 ^a^
2,3-dimethylpyrazine	1348	1356	nutty, cocoa	137 ± 8 ^b^	143 ± 10 ^b^	213 ± 19 ^a^	218 ± 14 ^a^	247 ± 13 ^a^
*Furans*								
furfural	1485	1499	almond-like	27,872 ± 1390 ^a^	23,179 ± 1833 ^b^	18,464 ± 799 ^c^	18,203 ± 1338 ^c^	17,712 ± 1008 ^c^
furfuryl alcohol	1669	1660	burnt	8488 ± 424 ^a^	6135 ± 368 ^b^	3107 ± 155 ^c^	2956 ± 89 ^c^	3196 ± 320 ^c^

^a^ Retention indexes from literature: pherobase.org for DB-WAX. ^b^ Calculated retention index values relatively to the n-alkane ladder. nd—not detected in analyzed samples. Values are means ± standard deviation (n = 3). Values in each column with different small superscript letters are significantly different (*p* < 0.05).

**Table 4:** Correlation between the rutin, TPC contents, antioxidant capacity (measured by DPPH and OxHLIA assays) with volatile compounds contents in rye-buckwheat biscuits.

Volatile Compounds	Rutin	TPC	DPPH	OxHLIA
Methylpyrazine	0.91	0.88	0.78	0.76
Ethylpyrazine	0.93	0.94	0.98	0.98
2,3-dimethylpyrazine	0.81	0.85	0.88	0.84
2,5-dimethypyrazine	0.82	0.86	0.81	0.84
2,6-dimethylpyrazine	0.86	0.86	0.81	0.82
Furfural	−0.66	−0.70	−0.84	−0.85
furfuryl alcohol	−0.61	−0.65	−0.79	−0.80
Hexanal	−0.77	−0.81	−0.88	−0.86

**Table 5:** Rye-buckwheat biscuit formulas with increasing rutin content.

Ingredients	CB	RBB	RBB-L	RBB-M	RBB-H
Rye flour type 720 [g]	100	70	70	70	70
Light buckwheat flour [g]	-	30	30	30	30
Buckwheat honey [g]	50	50	50	50	50
Sugar [g]	20	20	20	20	20
Baking powder [g]	3	3	3	3	3
Butter [g]	25	25	25	25	25
Rutin [mg]	0	0	10	50	100

Abbreviations: CB—control biscuits made of rye flour, RBB—rye-buckwheat biscuits made of rye and light buckwheat flour (70:30, *w/w*), RBB-L—rye-buckwheat biscuits made of rye and light buckwheat flour with low rutin addition (5 mg rutin/100 g of product), RBB-M—rye-buckwheat biscuits made of rye and light buckwheat flour with medium rutin addition (25 mg rutin/100 g of product), RBB-H—rye-buckwheat biscuits made of rye and light buckwheat flour with high rutin addition (50 mg rutin/100 g of product).
